# Memory-enhancing activities of the aqueous extract of *Albizia adianthifolia* leaves in the 6-hydroxydopamine-lesion rodent model of Parkinson’s disease

**DOI:** 10.1186/1472-6882-14-142

**Published:** 2014-04-30

**Authors:** Galba Jean Beppe, Alain Bertrand Dongmo, Harquin Simplice Foyet, Nolé Tsabang, Zenovia Olteanu, Oana Cioanca, Monica Hancianu, Théophile Dimo, Lucian Hritcu

**Affiliations:** 1 Laboratory of Animal Physiology, University of Yaoundé I, Yaounde, Cameroon; 2 Department of Animal Biology and Physiology, University of Douala, Douala, Cameroon; 3 Department of Agriculture, Cattle farming and Derived products, High Institute of the Sahel, University of Maroua, Maroua, Cameroon; 4 Department of Biology, Alexandru Ioan Cuza University, Iasi, Romania; 5 Faculty of Pharmacy, University of Medicine and Pharmacy “Gr. T. Popa”, Iasi, Romania; 6 Centre for Research on Medicinal Plants and Traditional Medicine, Institute of Medical Research and Medicinal Plant Studies, Yaoundé, Cameroon

**Keywords:** *Albizia adianthifolia* extract, Antioxidant activity, 6-hydroxydopamine-lesion rat, Memory, Parkinson disease

## Abstract

**Background:**

*Albizia adianthifolia* (Schumach.) W. Wright (Fabaceae) is a traditional herb largely used in the African traditional medicine as analgesic, purgative, anti-inflammatory, antioxidant, antimicrobial and memory-enhancer drug. This study was undertaken in order to evaluate the possible cognitive-enhancing and antioxidative effects of the aqueous extract of *A. adianthifolia* leaves in the 6-hydroxydopamine-lesion rodent model of Parkinson’s disease.

**Methods:**

The effect of the aqueous extract of *A. adianthifolia* leaves (150 and 300 mg/kg, orally, daily, for 21 days) on spatial memory performance was assessed using Y-maze and radial arm-maze tasks, as animal models of spatial memory. Pergolide - induced rotational behavior test was employed to validate unilateral damage to dopamine nigrostriatal neurons. Also, *in vitro* antioxidant activity was assessed through the estimation of total flavonoid and total phenolic contents along with determination of free radical scavenging activity. Statistical analyses were performed using two-way analysis of variance (ANOVA). Significant differences were determined by Tukey’s *post hoc* test. *F* values for which p < 0.05 were regarded as statistically significant. Pearson’s correlation coefficient and regression analysis were used in order to evaluate the association between behavioral parameters and net rotations in rotational behavior test.

**Results:**

The 6-OHDA-treated rats exhibited the following: decrease of spontaneous alternations percentage within Y-maze task and increase of working memory errors and reference memory errors within radial arm maze task. Administration of the aqueous extract of *A. adianthifolia* leaves significantly improved these parameters, suggesting positive effects on spatial memory formation. Also, the aqueous extract of *A. adianthifolia* leaves showed potent *in vitro* antioxidant activity. Furthermore, *in vivo* evaluation, the aqueous extract of *A. adianthifolia* leaves attenuated the contralateral rotational asymmetry observed by pergolide challenge in 6-OHDA-treated rats.

**Conclusions:**

Taken together, our results suggest that the aqueous extract of *A. adianthifolia* leaves possesses antioxidant potential and might provide an opportunity for management neurological abnormalities in Parkinson’s disease conditions.

## Background

Parkinson’s disease (PD), the second most common neurodegenerative disorder after Alzheimer’s disease (AD), is characterized by motor and behavioral disturbances that include a resting tremor, postural instability and bradykinesia [1]. PD affects approximately 2% of the population, although incidence varies across age, gender and race [2].

PD is often complicated by a variety of cognitive symptoms that range from isolated memory and thinking problems to severe dementia. While the motor symptoms of PD are well-known (tremor, rigidity, slowness of movement, imbalance), the commonly seen deficits in memory, attention, problem-solving, and language are less understood. Studies have shown that over 50% of people with PD experience some form of cognitive impairment. About 20% have more substantial cognitive impairment [3]. Memory problems in PD are typically milder than in Alzheimer’s disease. In PD, the person may have difficulty concentrating, learning new information and recalling names [3]. Non-motor symptoms in advanced stages of PD such as depression, dementia, sleep abnormalities and autonomic failure are probably the consequence of degeneration of both dopaminergic and non-dopaminergic systems, which still lacks efficacious treatments at present [4].

Although the etiology of the neurodegenerative processes found in PD is not completely understood, it is suggested that a state of oxidative imbalance is triggered by one or more factors, among which are brain aging, genetic predisposition, mitochondrial dysfunction, free radical production and environmental toxins [5-8].

Neuropathological evidence from both human and experimental models of PD firmly supports a significant role for oxidative stress in the death of dopaminergic (DA) neurons in the substantia nigra (SN) [9]. Although no model to date has been able to recapitulate all the pathological features of PD, the genetic or neurotoxic animal models of PD have contributed much to our understanding of human PD [10,11]. Neurotoxins-based models of PD have a long history and represent the most important models while genetic animal models have failed to recapitulate the key neurobehavioral or pathological features of PD [10,11].

6-hydroxydopamine (6-OHDA), 1-methyl-4-phenyl-1,2,3,6-tetrahydropyridine (MPTP) and rotenone, are the most successful agents so far to mimic parkinsonism *in vitro* and *in vivo*. 6-OHDA is taken up by the dopamine transporter (DAT) and it then generates free radicals [12]. Extensive study of these models has defined important cellular actors of cell death including oxidative stress, mitochondrial dysfunction, excitotoxicity, neuroinflammation and nitric oxide which is presumably critical of the nigral degeneration [13,14]. Furthermore, the neurotoxic models can serve as valuable tools for the assessment of efficacy and side-effects of symptomatic treatments of PD and have offered a basis for the development of novel therapeutic strategies [15].

Discovery of new drugs from traditional medicine is not a new phenomenon. Current researches are focusing on finding therapies, preferentially from natural products which could help in preventing/delaying the ongoing neurodegeneration in PD [16,17]. Research confirms that several medicinal herbs-based extracts increase redox/antioxidative abilities of the body and can effectively slow the progression of PD [3,17,18].

Among the plants used in traditional medicine, *Albizia adianthifolia* (Schumach.) W. Wright (Fabaceae) is commonly used in Cameroon as a remedy. In fact, it grows in most African countries. Its use in traditional medicine varies from one country to another. The sap is applied on the eye to treat river blindness and conjunctivitis, used as decoction, leaves treat respiratory diseases and have analgesic proprieties. An infusion or decoction of the bark is used to treat scabies and other skin diseases. A decoction of the leaves is administered as a purgative, as an analgesic and against inflammation. In Central and West Africa, this plant is used for the treatment of skin diseases, bronchitis, tapeworm, headaches and sinusitis [19,20].

Aqueous and ethanol extracts of *A. adianthifolia* (Schumach.) W. Wright (stem bark) used in southern Africa to treat memory loss and Alzheimer’s disease, have been screened for acetylcholinesterase inhibitory activity [21]. The root ethanolic extract of this plant showed *in vitro* immunomodulatory activity on the Jurkart T cell [22]. Kim et al. [23] reported that the aqueous extract of *A. julibrissin* had anxiolytic-like effects in rats as assessed using the elevated plus-maze test. Also, a recent study indicated that julibroside C1 extracted from *A. julibrissin* stem bark produced potent anxiolytic-like effects in mice [24]. Aurantiamide acetate was the most active compound isolated from the stem bark of *A. adianthifolia* through antioxidant activity (DPPH) and trolox equivalent antioxidant capacity (TEAC) assays were used to detect the antioxidant activity EC50 values 9.51 μg/ml and 78.81 μg/ml, respectively. The bark extracts of *A. lebbeck* possess free radical scavenging activity against 1,1-diphenyl-2-picrylhydrazyl radical (DPPH) and reducing power assays. Their results on DPPH free radical scavenging at 1000 μg/ml indicated maximum antioxidant activity of 91.82% and 90.08%, respectively. Ethanolic extract of *A. procera* showed strong scavenging activity against free radicals compared to various standards. These *in-vitro* assays indicate that these plant extracts are a good source of natural antioxidants, which might be helpful in preventing the progress of various oxidative stresses [25], with relevance for Parkinson’s disease conditions.

Despite extensive knowledge about the effects of *Albizia* species extracts, there is no study clarifying the possible cognitive-enhancing and antioxidant potentials of *A. adianthifolia* leaves extract in animal models of PD.

In this way, the present study aims to investigate the possible antioxidant activity and behavioral recovery following chronic administration of the aqueous extract of *A. adianthifolia* leaves using a unilateral 6-OHDA-lesion rat model of PD.

## Methods

### Plant material and plant extract

*Albizia adianthifolia* leaves were collected in Elounden, near Yaoundé (Cameroon) in June 2010 and identified by Dr. Nolé Tsabang at the National Herbarium Yaoundé where a voucher specimen (N° HNC 29997) was registered and deposited for ready reference. *A. adianthifolia* leaves were air dried and pulverized into fine powder. Five hundred grams of pulverized sample material was macerated in 5 L of distilled water for 48 h at room temperature and then the mixture was filtered through Whatman filter paper no. 3. The aqueous extract was then lyophilized to obtain powder used for our various tests. The dried extract was dissolved in distilled water and administered by gastric gavage to animals at the doses of 150 and 300 mg/kg body weight.

### HPLC (LC/DAD) analysis

**Figure 1:**
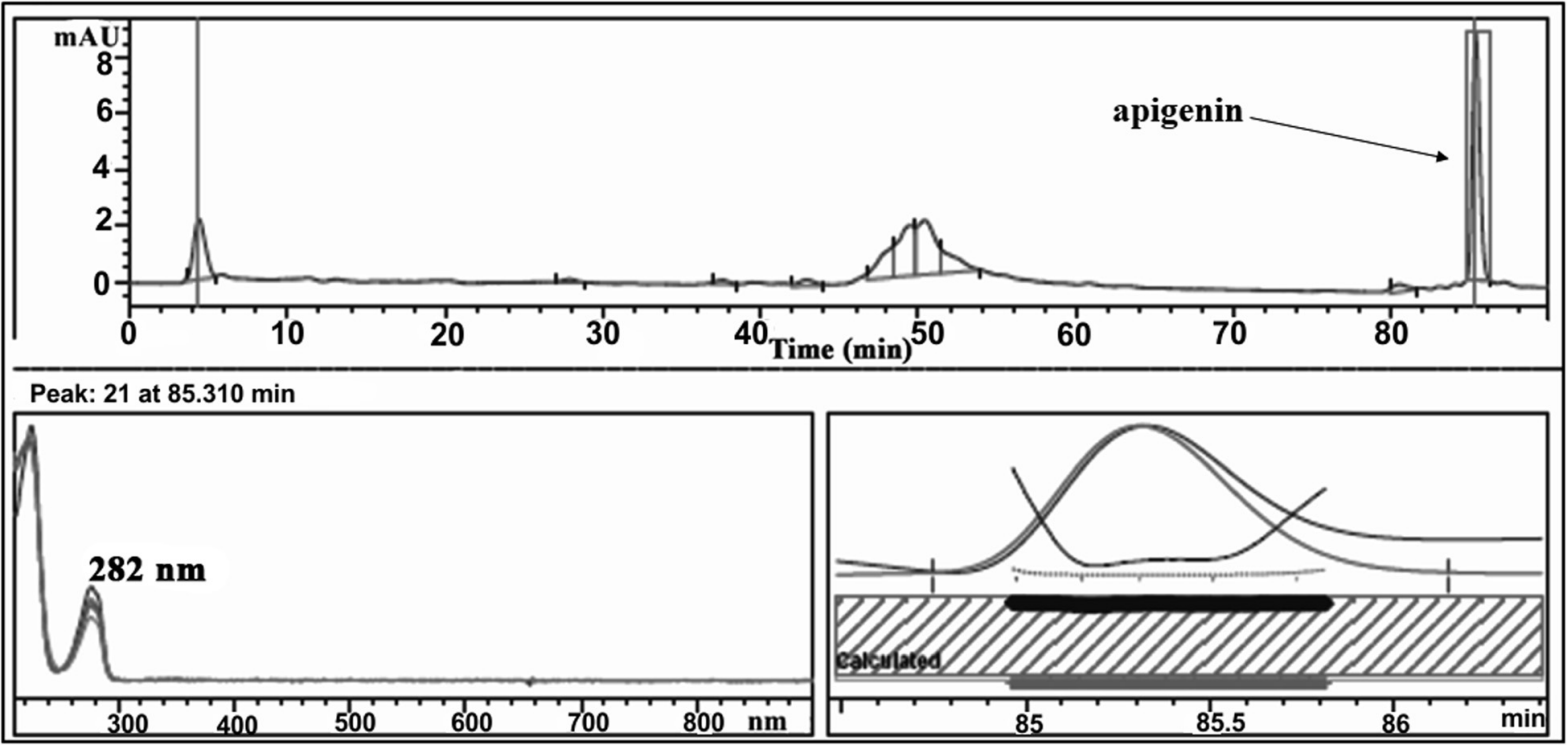
**HPLC data for apigenin isolated from the the aqueous extract *of A. adianthifolia *** **leaves.**

To identify the main compounds of the aqueous extract of *A. adianthifolia* leaves an Agilent 1200 HPLC system (Agilent Technologies, Palo Alto, CA, USA) and photodiode array detector (DAD) coupled with an analytical workstation were used. The working conditions are: Agilent Zorbax Eclipse XDB-C18 column (4.6 × 150 mm, 5 μm); column temperature: 40°C; detection wavelength: 203, 254, 282, 326 and 521 nm; flow rate: 1 mL/min; gradient elution: acetonitrile (solvent A) and water (solvent B); the initial conditions were 2% A and 98% B; the linear gradient programme was of 2-14-20-30-25% solvent A at 0-20-40-50-60 min, after which we switched back to the initial conditions; sample injection (20 μL) was performed by an autosampler programme. As standards we used caffeic, chlorogenic, ferulic, and rosmarinic acids, the flavonoids: apigenin, apigenin 7-O-glucoside, hyperoside, luteolin, luteolin 7-O-glucoside, rutin and quercetin (LG Standards). To generate the calibration curve, the standard stock solutions were diluted with methanol and analyzed in the same conditions. HPLC data revealed that among flavonoids, only apigenin was identified and quantified. Nevertheless, the levels of flavonoid compounds taken separately were low, this being the main characteristic of the sample (Figure 1).

### Animals

40 male Wistar rats weighing 350 ± 50 g at the start of the experiment were used. The animals were housed in a temperature and light-controlled room (22°C, a 12-h cycle starting at 08:00 h) and were fed and allowed to drink water *ad libitum*. Rats were treated in accordance with the guidelines of animal bioethics from the Act on Animal Experimentation and Animal Health and Welfare from Romania and all procedures were in compliance with Directive 2010/63/EU of the European Parliament and of the Council of 22 September 2010 on the protection of animals used for scientific purposes. This study was approved by the Ethics Committee of Alexandru Ioan Cuza University of Iasi, Romania and also, efforts were made to minimize animal suffering and to reduce the number of animal used.

### In vitro experimental test

#### Estimation of total flavonoid content

The aluminum chloride colorimetric method was modified from the previous procedure reported [26]. A 0.01% rutoside solution was used to make the calibration curve. 5 mL of the methanol extract solution (3 g lyophilized powder/100 g solution) was mixed with 3 mL of 2.5% aluminum chloride and 17 mL of methanol. After incubation at room temperature for 45 min, the absorbance of the reaction mixture was measured at 430 nm using a Shimadzu UV-1700 spectrophotometer. The amount of 2.5% aluminum chloride was substituted by the same amount of methanol in blank. The total flavonoid content was expressed as mg rutoside per gram of lyophilized powder. Each assay was repeated trice and the results, recorded as mean of the triplicated experiments.

### Estimation of total phenolic content

The total phenolic content of the lyophilized powder was determined by the Folin-Ciocalteu reagent [27] with modifications. The calibration curve was prepared using a 0.1% gallic acid solution. 40 μL extract solution (30 mg/ml) was mixed with 3160 μL of distilled water and 200 μL of Folin-Ciocalteu reagent. After 5 min, 600 μL of 20% sodium carbonate solution was mixed. The mixture was kept for incubation at room temperature for 2 h and the absorbance was measured at 765 nm using a Shimadzu UV-1700 spectrophotometer. The total phenolic content was expressed as mg gallic acid per gram of lyophilized powder. Each assay was repeated trice and the results, recorded as mean of the triplicated experiments.

### Determination of free radical scavenging activity

The free radical scavenging activity of the lyophilized powder was determined using the stable radical DPPH (2,4-dinitrophenyl-1-picryl hydrazyl) method as previously described [28]. 200 μL of the tested sample were placed in test tubes and 2 mL of freshly prepared DPPH solution (60 μM) in methanol was added in each test tube and mixed. 30 min later, the absorbance was measured at 517 nm (Shimadzu UV-1700 spectrophotometer). The capability to scavenge the DPPH radical was calculated, using the following equation:

$$\mathrm{DPPH}\;\mathrm{scavenged}\;\left(\%\right)=\left[\left(\;\mathrm{Ac}-\mathrm{At}\right)/\mathrm{Ac}\right]\times 100$$

Where Ac is the absorbance of the control reaction and At is the absorbance in presence of the plant samples. For the antioxidant activity determination we used a 3 mg/mL methanol extract solution. Each assay was repeated trice and the results, recorded as mean of the triplicated experiments.

### In vivo experiments

#### Neurosurgery

All surgical procedures were conducted under aseptic conditions, under sodium pentobarbital (50 mg/kg b.w., i.p., Sigma-Aldrich, Germany) anesthesia. Rats were mounted in the stereotaxic apparatus with the nose oriented 11° below horizontal zero plane. Thereafter, the animals received a right-unilaterally intranigral injections of 5 μL of 0.9% saline containing 2.5 μg/ μL 6-OHDA (free base) and 0.2% ascorbic acid (w/v) at a rate of 1 μL/min at the following coordinates: 5.5 mm posterior to bregma; 2.0 mm lateral to the midline; 7.4 mm ventral to the surface of the cortex, according to the stereotaxic atlas [29]. All rats sustaining 6-OHDA lesion were pretreated with desipramine (25 mg/kg, i.p. in saline) 30 min before anesthesia in order to protect noradrenergic system. The rats in the control group (sham-operated) were given 5 μL of 0.9% saline - 0.2% ascorbic acid administered in a similar manner as the solution containing 6-OHDA.

### Drug administration

The rats were divided into 4 groups (10 per group): (1) control group (sham-operated received distilled water treatment); (2) 6-OHDA-lesioned group, as negative control, received distilled water treatment; (3) 6-OHDA-lesioned group received 150 mg/kg of the aqueous extract of *A. adianthifolia* leaves treatment (6-OHDA + AE (150 mg/kg)); and (4) 6-OHDA-lesioned group received 300 mg/kg of the aqueous extract of *A. adianthifolia* leaves treatment (6-OHDA + AE (300 mg/kg)). The administration of the distilled water and the aqueous extract was performed by gastric gavage with biomedical needles (length 7.62 cm, ball diameter 4 mm, straight). The volume administered was 10 ml/kg of body weight, daily, for 21 consecutive days after neurosurgery.

### Rotational behavior

The animals were tested for rotational behavior by pergolide (0.5 mg/kg, b.w., s.c., Sigma-Aldrich, Germany) 1 week after the 6-OHDA injection. The drug pergolide functions as a dopamine receptor agonist for D_2_ and D_1_ receptors and as a ligand to serotonin 5-HT_1A_, 5-HT_1B_, 5-HT_2A_, 5-HT_2B_, and 5-HT_2C_ receptors [30,31]. Also, it was argued that pergolide induced contralateral rotation in animals with striatal lesion after systemic administration [32]. Briefly, 1 min after pergolide injection, full rotations were counted in a cylindrical container (a diameter of 33 cm and a height of 35 cm) at 10 min intervals for 60 min in a quiet isolated room. Rotations in the ipsilateral and contralateral directions were counted separately and the analyses were based on the net scores (contralateral minus ipsilateral rotations) recorded for 60 min [33].

### Y-maze task

Short-term memory was assessed by spontaneous alternation behavior in the Y-maze task. The Y-maze used in the present study consisted of three arms (35 cm long, 25 cm high and 10 cm wide) and an equilateral triangular central area. 30 min after the aqueous extract of *A. adianthifolia* leaves administration, rats were placed at the end of one arm and allowed to move freely through the maze for 8 min. An arm entry was counted when the hind paws of the rat were completely within the arm. Spontaneous alternation behavior was defined as entry into all three arms on consecutive choices. The number of maximum spontaneous alternation behaviors was then the total number of arms entered minus 2 and percent spontaneous alternation was calculated as (actual alternations/maximum alternations) × 100. The maze was cleaned with a 10% ethanol solution and dried with a cloth before the next animal was tested. Spontaneous alternation behavior is considered to reflect spatial working memory, which is a form of short-term memory [34,35].

### Radial 8 arm-maze task

The radial 8 arm-maze used in the present study consisted of 8 arms, numbered from 1 to 8 (48 × 12 cm), extending radially from a central area (32 cm in diameter). The apparatus was placed 40 cm above the floor, and surrounded by various extramaze visual cues placed at the same position during the study. At the end of each arm there was a food cup that had a single 50 mg food pellet. Prior to the performance of the maze task, the animals were kept on restricted diet and body weight was maintained at 85% of their free-feeding weight over a week period, with water being available ad libitum. Before the actual training began, three or four rats were simultaneously placed in the radial maze and allowed to explore for 5 minutes and take food freely. The food was initially available throughout the maze, but was gradually restricted to the food cup. The animals were trained for 4 days to run to the end of the arms and consume the bait. To evaluate basal activity of rats in radial 8 arm-maze, the rats were given 1 training trial per day to run to the end of the arms and consume the bait. The training trial continued until all the 5 baits had been consumed or until 5 minutes has elapsed. After adaptation, all rats were trained with 1 trial per day. Briefly, 30 min after the aqueous extract of *A. adianthifolia* leaves administration, each animal was placed individually in the center of the maze and subjected to working and reference memory tasks, in which same 5 arms (no. 1, 2, 4, 5 and 7), were baited for each daily training trial. The other 3 arms (no. 3, 6 and 8) were never baited. An arm entry was counted when all four limbs of the rat were within an arm. Measures were made of the number of working memory errors (entering an arm containing food, but previously entered), reference memory errors (entering an arm that was not baited). The maze was cleaned with a 10% ethanol solution and dried with a cloth before the next animal was tested. Reference memory is regarded as a long-term memory for information that remains constant over repeated trials (memory for the positions of baited arms), whereas working memory is considered a short-time memory in which the information to be remembered changes in every trial (memory for the positions of arms that had already been visited in each trial) [35,36].

### Histological control

At the end of the experiment, all animals were killed with overdose of sodium pentobarbital (100 mg/kg b.w. i.p., Sigma-Aldrich, Germany) followed by the transcardial infusion of 0.9% saline and a 10% formalin solution. The brain were removed and placed in a 30% sucrose/formalin solution. The brain were frozen and cut into coronal sections (50 μm) using a freezing microtome and stained with crezyl violet for verification of the point of the syringe needle. Only experimental data from lesions correctly located in the substantia nigra (SN) were used for statistical analysis.

### Statistical analysis

The animal’s behavioral activities in Y-maze and radial 8 arm-maze tasks were statistically analyzed by two-way analysis of variance (ANOVA) using XLSTAT version 2012. 1.01 software, Addinsoft. In the spontaneous alternation experiments within Y-maze task, one sample t-test was used for comparison of the alternation to chance level (50%). In order to evaluate differences between groups in radial arm-maze task, separate repeated-measures ANOVA were calculated on number of working memory errors and number of reference memory errors with group (Control, 6-OHDA, 6-OHDA + AE (150 mg/kg) and (6-OHDA + AE (300 mg/kg)) as between-subject factor and days (1 to 7) as within-subjects factors. All results are expressed as mean ± S.E.M. F values for which p < 0.05 were regarded as statistically significant. Significant differences were determined by Tukey’s *post hoc* test. Pearson’s correlation coefficient and regression analysis were used in order to evaluate the association between behavioral parameters and net rotations in rotational behavior test.

## Results

### Histological verification

After 6-OHDA-lesions, the rats recovered quickly and gained weight by first week. In the majority of SN-lesioned rats the point of the syringe needle was positioned in the central part of the SN and the lesions extended to a part of adjacent structures including substantia nigra pars reticulata, without any significant damage.

### Total flavonoids, total phenolic content and free radical-scavenging capacity of the A. adianthifolia leaves

The methanol extracts of the tested *A. adianthifolia* leaves exhibited high content of phenols and high radical scavenging property (DPPH scavenged %). Additionally, *A. adianthifolia* leaves present low level of the flavonoids. The results strongly suggest that phenolics and flavonoids are important components of the *A. adianthifolia* leaves and this could explain their high radical scavenging activity (Table 1).

**Table 1:** **Total flavonoids, total phenolic content and free-scavenging capacity of the extract of *Albizia adianthifolia *** **leaves**

	**Phenols**	**Flavonoids**	**DPPH**
	**(mg gallic acid/g lyophilized powder)**	**(mg rutoside/g lyophilized powder)**	**scavenged%**
*Albizia adianthifolia* leaves extract	30.172 ± 0.863	0.525 ± 0.001	58.189 ± 0.601

### Effect pergolide on rotational behavior

**Figure 2:**
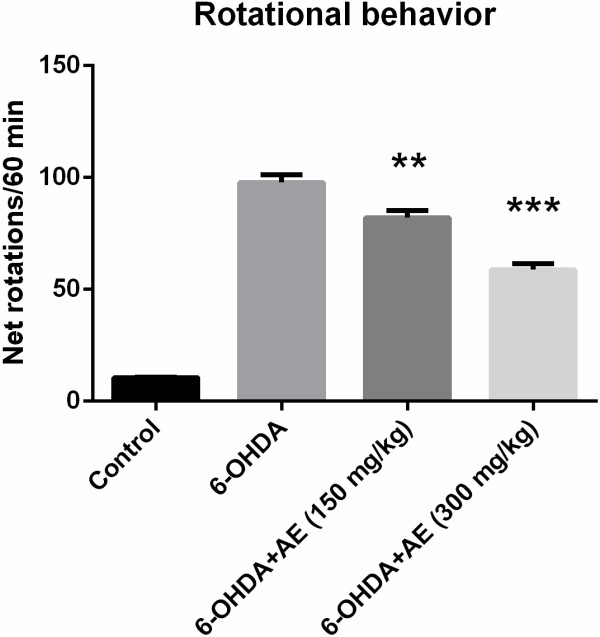
**Protective effects of the aqueous extract of *A. adianthifolia *leaves (150 and 300 mg/kg) on pergolide (0.5 mg/kg, b.w., s.c.)-induced rotational behavior in 6-OHDA-lesioned rats.** 1 week after 6-OHDA injection, the number of net rotation contralateral to the lesion side was increased. However, the administration of the aqueous extract of *A. adianthifolia* leaves restored the decrease in the number of net rotation. Values are means ± S.E.M. (n = 10 animals per group), **p < 0.001, ***p < 0.0001 vs. 6-OHDA alone treated-group.

Pergolide-induced rotational behavior was analyzed to assess the unilateral degeneration of the dopamine nigrostriatal neurons. Control group did not show any significant any significant bias in turning behavior after receiving pergolide injection. In contrast, rats exhibited contralateral rotational behavior following pergolide challenge in 1 week after the unilateral administration of 6-OHDA into the substantia nigra. In the rotational behavioral test, ANOVA revealed an attenuation of asymmetric motor behavior (F(3,36) = 20.03, p < 0.0001) in experimental animals treated with the aqueous extract of *A. adianthifolia* leaves (150 and 300 mg/kg) in a dose-dependent manner as compared to control group (Figure 2). Additionally, Tukey’s *post hoc* analysis revealed a significant difference between the control and 6-OHDA groups (p < 0.0001), control and 6-OHDA + AE (150 mg/kg) groups (p < 0.0001), control and 6-OHDA + AE (300 mg/kg) groups (p < 0.0001), 6-OHDA and 6-OHDA + AE (150 mg/kg) groups (p < 0.001), 6-OHDA and 6-OHDA + AE (300 mg/kg) groups (p < 0.0001) and 6-OHDA + AE (150 mg/kg) and 6-OHDA + AE (300 mg/kg) groups (p < 0.0001) (Figure 2).

### Effect of A. adianthifolia extract on spatial memory in Y-maze task

**Figure 3:**
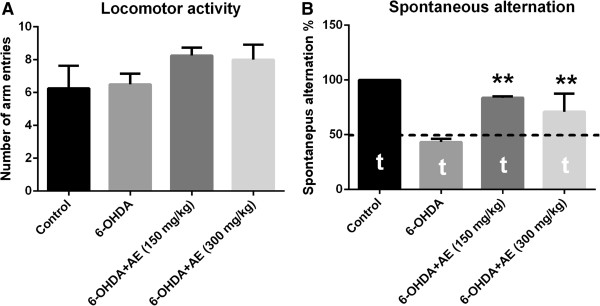
**Effects of the aqueous extract of *A. adianthifolia *leaves (150 and 300 mg/kg) on spontaneous alternations% (B) and number of arm entries (A) in the 6-OHDA-treated rats.** Values are means ± S.E.M. (n = 10 animals per group), **p < 0.001 vs. 6-OHDA alone treated-group.

In the Y-maze task, ANOVA revealed a significant increase of spatial memory performance in 6-OHDA-lesioned groups treated with low-and high-doses (150 and 300 mg/kg) of the aqueous extract of *A. adianthifolia* leaves (F(3,36) = 8.21, p < 0.001) (Figure 3B), indicated by an increase of the spontaneous alternation percentage compared to 6-OHDA alone-treated group, suggesting significant effects on short-term memory. Additionally, Tukey’s *post hoc* analysis revealed a significant difference between the control and 6-OHDA groups (p < 0.0001), control and 6-OHDA + AE (150 mg/kg) groups (p < 0.0001), 6-OHDA and 6-OHDA + AE (150 mg/kg) groups (p < 0.0001) and 6-OHDA and 6-OHDA + AE (300 mg/kg) groups (p < 0.001), indicating that the aqueous extract significantly improved spatial working memory (Figure 3B). Furthermore, spontaneous alternation percentage in control (t = 8.86, p = 0.001), 6-OHDA (t = 14.63, p = 0.001), 6-OHDA and 6-OHDA + AE (150 mg/kg) (t = 61.93, p = 0.0001) and 6-OHDA and 6-OHDA + AE (300 mg/kg) (t = 4.32, p = 0.05) groups were statistically different from the chance level (50%) (Figure 3B).

The changes in the spontaneous alternation percentage of both 6-OHDA and 6-OHDA + AE (150 mg/kg) and 6-OHDA and 6-OHDA + AE (300 mg/kg) groups are not related to the changes in locomotor activity, as evidenced in Y-maze task by the number of arm entries (Figure 3A).

### Effect of A. adianthifolia extract on spatial memory in radial 8 arm-maze task

To investigate whether the aqueous extract of *A. adianthifolia* leaves (150 and 300 mg/kg) affects spatial memory formation, the rats were further evaluated in the radial arm-maze task.

**Figure 4:**
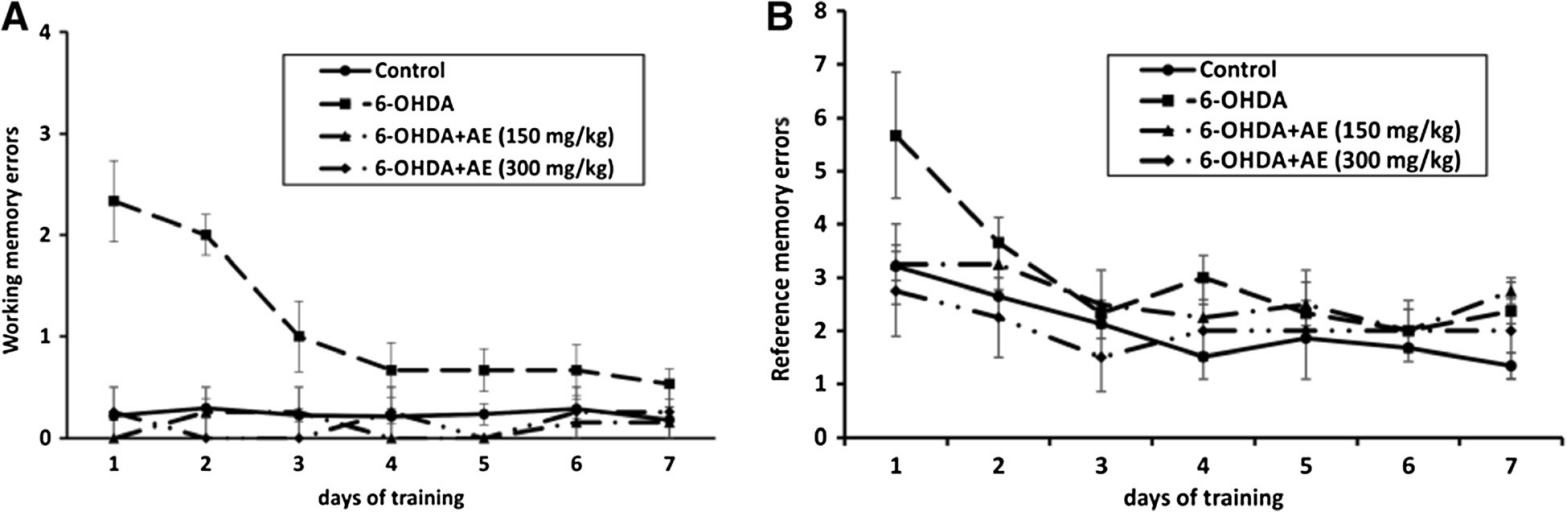
**Effects of the aqueous extract of *A. adianthifolia *leaves (150 and 300 mg/kg) on the working memory errors (A) and the reference memory errors (B) during 7 days training in radial arm-maze task.** Values are means ± S.E.M. (n = 10 animals per group).

For working memory errors, repeated-measures ANOVA revealed non-significant effects of time-group interaction (F(12,91) = 0.679, p > 0.05) (Figure 4A). Additionally, Tukey’s *post hoc* analysis revealed significant differences between the control and 6-OHDA groups (p < 0.001), 6-OHDA and 6-OHDA + AE (150 mg/kg) groups (p < 0.001) and 6-OHDA and 6-OHDA + AE (300 mg/kg) groups (p < 0.001) for working memory errors (Figure 4A), indicating that the aqueous extract of *A. adianthifolia* leaves significantly improved working memory during 7 days training in radial arm-maze task.

For reference memory errors, repeated-measures ANOVA revealed a significant time difference (F(6,91) = 4.957, p < 0.0001) (Figure 4B). Additionally, Tukey’s *post hoc* analysis revealed significant differences between the control and 6-OHDA groups (p < 0.01), control and 6-OHDA + AE (150 mg/kg) groups (p < 0.01) and 6-OHDA and 6-OHDA + AE (300 mg/kg) groups (p < 0.05) for reference memory errors (Figure 4B), indicating that the aqueous extract of *A. adianthifolia* leaves significantly improved long-term memory during 7 days training in radial arm-maze task.

**Figure 5:**
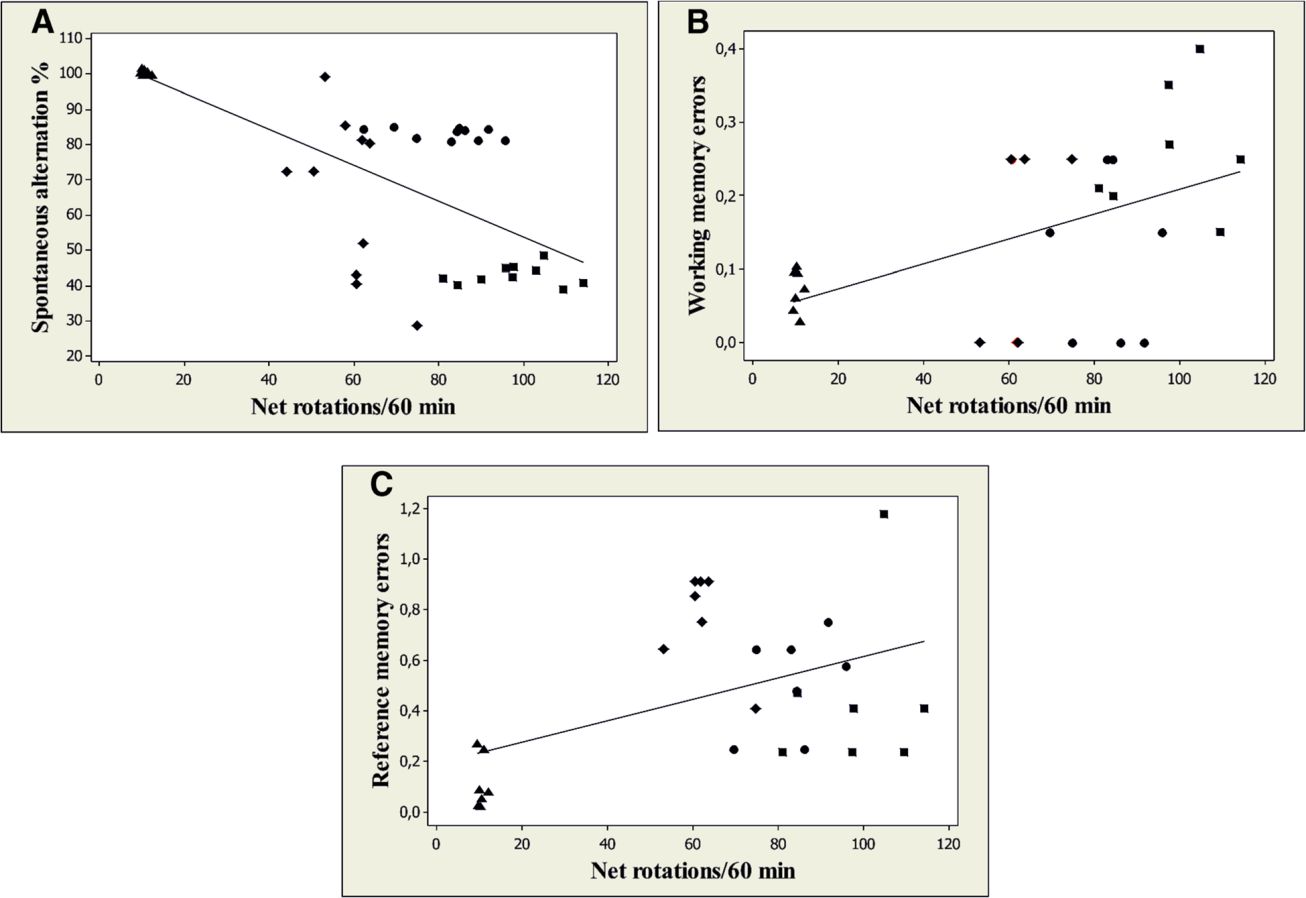
Correlation between the percentages of spontaneous alternation vs. net rotation (A), working memory errors vs. net rotation (B) and reference memory errors vs. net rotation (C) in control group (▲), 6-OHDA alone treated group (■), 6-OHDA + AE (150 mg/kg) group (●) and 6-OHDA + AE (300 mg/kg) group (♦).

More importantly, when linear regression was determined, a significant correlation between the percentages of spontaneous alternation vs. net rotation (n = 40, r = −0.723, p < 0.0001) (Figure 5A), working memory errors vs. net rotation (n = 40, r = 0.497, p < 0.001) (Figure 5B) and reference memory errors vs. net rotation (n = 40, r = 0.466, p < 0.01) (Figure 5C) was evidenced.

## Discussion

In recent years, much attention has been focused on the protective biochemical function of naturally present antioxidants in biological systems, and on the mechanisms of their action [37]. *A. adianthifolia* (Schumach.) W. Wright (Fabaceae) is used in Central and West Africa for the treatment of skin diseases, bronchitis, inflamed eyes, tapeworm, headaches and sinusitis [19,20]. The roots of *A. adianthifolia* are used for improving memory in Venda Region, Southern Africa [38]. However, the scientific bases of the use of this plant have not yet clearly established. The antioxidant and neuroprotective activity of this herb extract was determined in the present study.

Phenolic and nitrogenous compounds are known to be potent antioxidants due to their ability to scavenge free radicals and active oxygen species such as singlet oxygen, superoxide anion radical and hydroxyl radicals [39-41]. Our results showed that *A. adianthifolia* extract possesses high contents of phenols. The presence of such compounds could be responsible for the strongest *in vitro* antioxidant activity found in the aqueous extract of *A. adianthifolia* leaves. Theses observed *in vitro* activities suggest that this plant extract could exert protective effects also *in vivo* against oxidative and free radical injuries occurring in different pathological conditions, including neurodegenerative diseases.

It is well known that the substantia nigra is the area of the brain that is most affected in PD but other brain areas are also affected. The hippocampus is the brain area that plays an important role in spatial memory [42,43], therefore we determined the cognitive performance of rats using the Y-maze and radial arm-maze tests, as animal models of spatial memory. Moreover, it is well known that hippocampal formation is involved in learning and memory, and it was reported that the hippocampus plays an important role in processing, and remembering spatial and contextual information [44].

Intranigral injection of the 6-OHDA interferes with memory function subsequently causes impairment of spatial memory within the Y-maze and radial arm-maze tasks, in accordance with previous investigation using rats [3,45,46]. In the *in vivo* studies, the unilateral 6-OHDA-lesion rat model was used to determinate the behavioral recovery following 7 days of the aqueous extract of *A. adianthifolia* leaves administration.

The pergolide results confirm the impairment of the dopaminergic system. It has been shown that the dentate gyrus of the hippocampus received the dopaminergic projection from the ventral tegmental area (A10) and the substantia nigra (A9) [46]. The dopaminergic connection to the hippocampus might be affected leading to cognitive deficits observed in the 6-OHDA-treated rats. Furthermore, our results suggest that the antioxidant activity of the aqueous extract of *A. adianthifolia* leaves contributed to observed spatial memory improvements.

In the present study we used two well-characterized hippocampus-dependent spatial memory tasks: Y-maze and radial arm-maze. Our results showed that the aqueous extract of *A. adianthifolia* leaves sustain memory formation in the 6- OHDA-lesion rodent model of PD.

The HPLC analysis determined that among flavones, the main component is apigenin, so this is probably the constituent responsible for the observed cognitive-enhancing effects in the 6-OHDA-lesion rodent model of PD. It is suggested that apigenin and related compounds stimulate adult neurogenesis *in vivo* and *in vitro*, by promoting neuronal differentiation and also promotes learning and memory performance in the Morris water task [47].

The Y-maze task is a specific and sensitive test of spatial recognition memory in rodents. The test relies on an innate tendency of rats to explore a novel environment [48]. The Y-maze used in this study involves no aversive stimuli and was considered suitable for evaluating memory. The specific part of the brain involved in the performance of this task include the hippocampus [49].

As shown in Figure 3, the aqueous extract of *A. adianthifolia* leaves (150 and 300 mg/kg) in 6-OHDA-treated rats, but especially the low dose (150 mg/kg), significantly improved short-term memory, as evidenced by the percentage of spontaneous alternation as compared to 6-OHDA alone-treated rats. This result suggests that both doses of the aqueous extract of *A. adianthifolia* leaves used in this study display an improved effect on acquisition of the short-term memory of the 6-OHDA-treated rats within the Y-maze task. However, no differences were observed between both doses of the aqueous extract of *A. adianthifolia* leaves on spatial working memory in the Y-maze task. Also, the aqueous extract of *A. adianthifolia* leaves increased both the locomotor activity as well as short-term memory in 6-OHDA-treated rats within the Y-maze task. This effect of the aqueous extract observed in short-term memory cannot be attributed exclusively to increase locomotor activity, because the percentage of spontaneous alternation was also improved and the effect of improving working and reference memory is also observed in radial arm-maze task. Therefore, the improvement of short-term memory observed in 6-OHDA rats treated with the aqueous extract is not an artifact due to the concomitant increase in motor activity.

In the behavioral neuroscience trial, radial 8 arm-maze (RAM) task is widely used [35,50]. These RAM tests are useful in evaluating the effect of drugs, stress and various other environmental factors on learning and memory [51]. Working memory and reference memory are the two variables that report the physiological status of the brain [51]. Thus, 6-OHDA rats treated with the aqueous extract (150 and 300 mg/kg) exhibited an improvement of working memory (Figure 4A) as compared to 6-OHDA alone-treated rats, during 7 days training in radial arm-maze task. On the other hand, both doses of the aqueous extract (150 and 300 mg/kg) significantly improved long-term memory of 6-OHDA-treated rats, explored by reference memory (Figure 4B) as compared to 6-OHDA alone-treated rats, during 7 days training in radial arm-maze task. These findings could suggest that the aqueous extract plays an important role in spatial memory formation, especially on working and reference memories. However, no significant differences were observed between both doses of the aqueous extract on working memory and reference memory in radial arm-maze task.

Moreover, we found a significant positive correlation between the percentages of spontaneous alternation vs. net rotation, working memory errors vs. net rotation and reference memory errors vs. net rotation when linear regression was determined. These results could suggest that increase of behavioral parameters in the Y-maze and radial arm-maze tasks along with the decrease of the contralateral rotational asymmetry observed by pergolide challenge in 6-OHDA-treated rats could be related to involvement of the aqueous extract of *A. adianthifolia* leaves in neuroprotection against 6-OHDA-induced contralateral rotational behavior following pergolide challenge.

Regarding the limitation of our study we can add that there is an indirect behavioral evidence (e.g. rotational behavior) that the lesion worked rather than neurochemistry of the striatum to confirm the size of lesion.

## Conclusions

In summary, this study demonstrated that the aqueous extract of *A. adianthifolia* leaves exerts memory-enhancing effects in the 6-OHDA-lesion rodent model of PD via its antioxidant effects. Consequently, the use of this aqueous extract as an adjuvant therapeutic agent for the treatment of the cognitive impairment in PD should be considered.

## Competing interest

The authors declare that they have no competing interest.

## Authors’ contributions

GJB, ZO, NT, OC, MH and LH carried out the study; LH, ABD, HSF, and TD designed the experiments. LH and GJB wrote the manuscript; LH supervised the work; GJB provided the aqueous extract of *A. adianthifolia* leaves; all authors read and approved the final manuscript.

## Pre-publication history

The pre-publication history for this paper can be accessed here:

http://www.biomedcentral.com/1472-6882/14/142/prepub
